# Brief speech samples reveal emotional states in daily life

**DOI:** 10.1093/pnasnexus/pgag263

**Published:** 2026-08-06

**Authors:** Timo K Koch, Gabriella M Harari, Samuel D Gosling, Zachariah Marrero, Ramona Schoedel, Markus Bühner, Clemens Stachl

**Affiliations:** Institute of Behavioral Science and Technology, University of St Gallen, St Gallen 9000, Switzerland; Department of Psychology, Ludwig–Maximilians–Universität München, Munich 80802, Germany; Department of Communication, Stanford University, Stanford, CA 94305, USA; Department of Communication, Stanford University, Stanford, CA 94305, USA; Department of Psychology, The University of Texas at Austin, Austin, TX 78712, USA; Department of Psychology, The University of Texas at Austin, Austin, TX 78712, USA; Department of Psychology, Ludwig–Maximilians–Universität München, Munich 80802, Germany; Department of Psychology, Charlotte Fresenius Hochschule, University of Psychology, Munich 80797, Germany; Department of Psychology, Ludwig–Maximilians–Universität München, Munich 80802, Germany; Institute of Behavioral Science and Technology, University of St Gallen, St Gallen 9000, Switzerland

**Keywords:** emotional states, speech, voice, smartphones, foundation models

## Abstract

Does our speech—“what” we say and “how” we say it—reveal how we subjectively feel in daily life? Speech and emotion are often thought to be linked, but most supporting evidence comes from controlled laboratory settings and focuses on expressed, enacted, or externally labeled emotions, leaving open the question of whether naturalistic speech reflects subjective emotional experience in daily life. To address this question, we extracted both modern foundation-model embeddings and established speech-analysis variables from brief, prompted smartphone recordings, including Linguistic Inquiry and Word Count features and prosodic descriptors, and used them as inputs to supervised machine learning models predicting concurrent self-reported emotional states (934 participants; 12,285 observations). Cross-validated models evaluated on unseen participants captured information about self-reported emotional states (contentment: median Spearman *ρ* = 0.37; sadness: *ρ* = 0.24; arousal: *ρ* = 0.35), with spoken content represented by foundation-model text embeddings carrying the strongest emotional signal. Interpretability analyses provided further insights into the linguistic characteristics of everyday emotional language. These findings provide large-scale evidence that brief speech samples contain information about subjective emotional states in daily life.

Significance statement Speech is widely assumed to reflect emotion, yet it remains unclear whether it reveals how people subjectively feel in daily life. Many speech-based emotion inference systems rely on this assumption despite being trained on acted or labeled speech from controlled settings rather than naturalistic expressions of lived experience. Using 12,285 brief, prompted, smartphone-captured speech samples from 934 individuals along with concurrently collected emotion self-reports, we tested whether speech supports inference of subjective emotional experience. The most consistent signal came from semantic content captured by foundation-model text embeddings, while traditional prosodic descriptors contributed little under naturalistic recording conditions. These results clarify the capacities and limits of speech-based emotion inference systems trained on naturalistic data, with implications for research and real-world applications.

## Introduction

Does our speech—“what” we say and “how” we say it—contain information about how we subjectively feel in daily life? Scholars have long assumed that both the semantic content and acoustic properties of speech carry emotional information (1–5), but most empirical evidence comes from controlled laboratory paradigms and focuses on expressed, enacted, or externally labeled emotions (6). As a result, it remains unclear whether naturalistic speech produced in daily life reflects emotional experience as it is lived, rather than how emotions are expressed or portrayed in the lab. This discrepancy is consequential for research because it bears on whether speech in everyday life tracks subjective emotional experience rather than merely observable emotional expression. At the same time, it has practical implications because automated speech-based emotion inference is increasingly used in settings, such as customer-service analytics and digital well-being tools. Clarifying whether naturalistic speech contains information about subjective emotional experience is critical for understanding what speech can reveal about how people feel outside the laboratory.

Previous research linking speech and emotion has largely fallen into two camps. First, controlled laboratory studies have shown that enacted or induced emotions are systematically associated with both prosodic features of speech (2, 4, 6, 7), such as pitch, rhythm, and intensity, and the semantic content of what people say (8, 9). Yet these paradigms primarily capture how emotions are “expressed” under constrained conditions and may therefore not inform how spontaneous speech reflects experienced emotion in daily life (4, 10). Second, a growing body of naturalistic studies has paired everyday speech with in situ self-reports of emotion, thereby addressing the question of whether speech in the wild reflects how speakers themselves feel. Across these studies, linguistic and acoustic approaches have generally yielded modest associations, with findings varying across emotional outcomes, speech representations, and analytic methods (11–14). Small samples and heterogeneous data collection designs have further limited the conclusions that can be drawn. Consequently, it remains unclear whether naturalistic speech reliably supports inferences about subjective emotional experience in daily contexts.

Recent advances in foundation models—large neural networks trained on extensive text and audio corpora—provide a new opportunity to revisit this question. By representing speech in high-dimensional embeddings (15), these models offer a stringent test of whether speech contains information about subjective emotional experience under naturalistic recording conditions. Foundation models have shown promise for inferring relatively stable trait differences from both spoken and written language (16, 17), but their utility for inferring subjective momentary emotional states from everyday speech remains largely unexplored. Smartphones provide a particularly valuable platform for this investigation because they enable large-scale collection of brief, in situ speech samples together with concurrent self-reports of emotion, thereby linking speech more directly to lived emotional experience in daily life (11, 12, 18, 19).

In the present work, we leverage these developments to provide a large-scale investigation of whether brief, prompted diary-style speech samples collected via smartphones contain information about people's subjective emotional states in daily life. Using foundation-model text and speech embeddings together with established linguistic and prosodic descriptors, we analyze 12,285 speech samples from 934 participants paired with emotion self-reports. Our aims are to test whether naturalistic speech carries information about subjective emotional experience in everyday life and to identify the linguistic properties through which it is expressed under real-world recording constraints.

## Results

Here, we report the cross-validated performance of supervised machine learning models predicting self-reported emotional states (i.e. contentment, sadness, and arousal) from brief, prompted, smartphone-collected speech samples. We summarize model performance as the median Spearman correlation (*ρ*) between self-reported and predicted emotion scores and a 95% resampling interval (2.5th and 97.5th percentiles). Because evaluation was blocked by participants, performance reflects generalization to unseen individuals. We focus on elastic-net models, given their superior average predictive performance over random forest models, parsimony, and ability to effectively reduce predictor variables. Detailed performance metrics for elastic net and random forest prediction models across variable sets and emotional states are available in Table S1.

### Brief speech samples contain information about subjective emotional states

**Figure 1:**
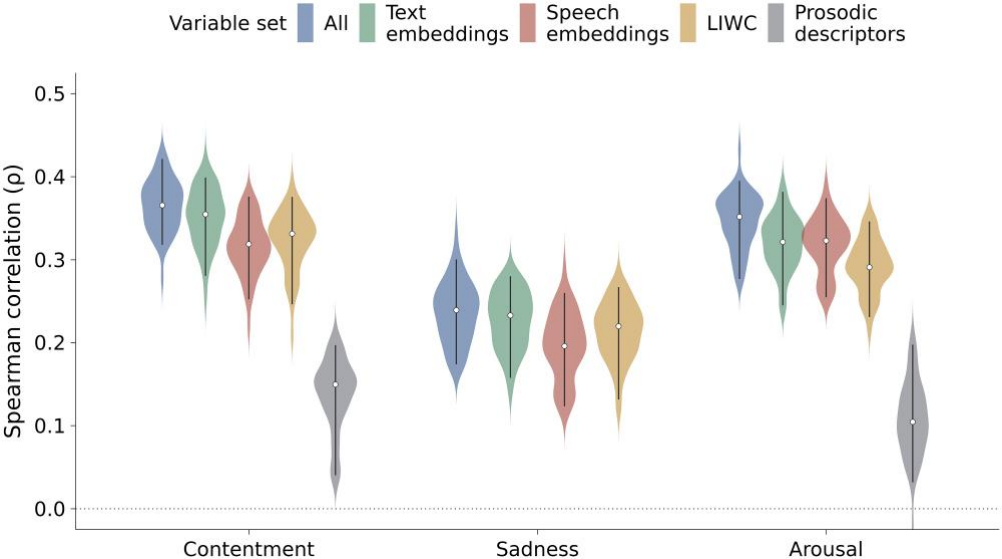
Out-of-sample prediction performance as measured by Spearman's *ρ* from a total of 50 resampling iterations of elastic-net models resulting from five times repeated 10-fold cross-validation (blocked by participants) per variable set and emotional state. Inside the violin plots, the white dot marks the median and the black line indicates the 2.5th and 97.5th percentiles (95% of the fold-wise performance values). Variable sets are text embeddings from a large language foundation model (RoBERTa), speech embeddings from a speech foundation model (wav2vec 2.0), word dictionaries from the LIWC-22, prosodic descriptors in the form of the eGeMAPS, and their combinations. For sadness, the elastic-net models trained on prosodic descriptors produced effectively constant predictions within folds and, as a result, Spearman correlations were undefined.

Overall, we find that prompted, diary-style speech samples collected with smartphones contained information about emotional states (Fig. 1) with the highest performance for contentment (median *ρ* = 0.37, 95% resampling interval = [0.32, 0.42]), followed by arousal (*ρ* = 0.35 [0.28, 0.40]) and sadness (*ρ* = 0.24 [0.17, 0.30]). Models trained on the spoken content of speech samples performed best among extracted variables, with text embeddings yielding the highest prediction performance overall across emotional states (*ρ* = 0.36 [0.28, 0.40] for contentment; *ρ* = 0.23 [0.16, 0.28] for sadness; *ρ* = 0.32 [0.25, 0.38] for arousal). Dictionary-based Linguistic Inquiry and Word Count (LIWC) variables reached slightly lower prediction levels (*ρ* = 0.33 [0.25, 0.38] for contentment; *ρ* = 0.22 [0.13, 0.27] for sadness; *ρ* = 0.29 [0.23, 0.35] for arousal). Speech embeddings reached similar performances (*ρ* = 0.32 [0.25, 0.38] for contentment; *ρ* = 0.20 [0.12, 0.26] for sadness; *ρ* = 0.32 [0.26, 0.37] for arousal). Finally, traditional prosodic descriptors carried the least predictive signal for self-reported emotional states under these naturalistic recording conditions (contentment: *ρ* = 0.15 [0.04, 0.20]; arousal: *ρ* = 0.11 [0.03, 0.20]) and yielded undefined performance estimates for sadness.^1^ Overall, models predicted contentment and arousal better on average than sadness. This lower performance of sadness may reflect the fact that it was reported at lower levels (*M* = 0.49, SD = 0.75) than other states (contentment: *M* = 1.65, SD = 0.86; arousal: *M* = 1.95, SD = 0.95; see Fig. S1 for their distributions), which may have limited the amount of generalizable sadness-specific signal available to the models. In addition, sadness may be less consistently expressed in naturalistic speech, potentially through subtle cues or even silence rather than clear semantic or prosodic markers, making it less likely to be captured in our recording setting (20).

After masking explicit emotion descriptors in the transcripts (e.g. “happy” or “angry”) to test how much content-based models relied on explicit verbalizations of emotional states, predictive performance decreased modestly. The decline was larger for models trained on LIWC categories (Δ*ρ* = 0.05–0.07) than for models trained on text embeddings (Δ*ρ* = 0.02–0.04), suggesting that LIWC models could rely more on explicit emotion verbalizations as reflected in the respective word categories, whereas embeddings could still capture contextual signal after masking (Table S1). Further, model performance, reflected in lower absolute prediction error, generally improved with voiced-speech duration up to ∼30 s, although the precise shape of this pattern varied across emotional states (Fig. S2). At longer durations, observations became increasingly sparse and prediction error estimates were less stable. Accordingly, the apparent pattern at higher durations should be interpreted cautiously. Sadness showed comparatively little variation across durations, likely reflecting its overall weaker predictability. Finally, the strong performance of embeddings was not merely an artifact of their higher dimensionality: even after applying principal component analysis (PCA) to reduce both text and speech embeddings to 100 components each, they retained predictive performance broadly comparable to or higher than that of LIWC and prosodic descriptors (Table S1).

### Linguistic characteristics of emotional speech

**Figure 2:**
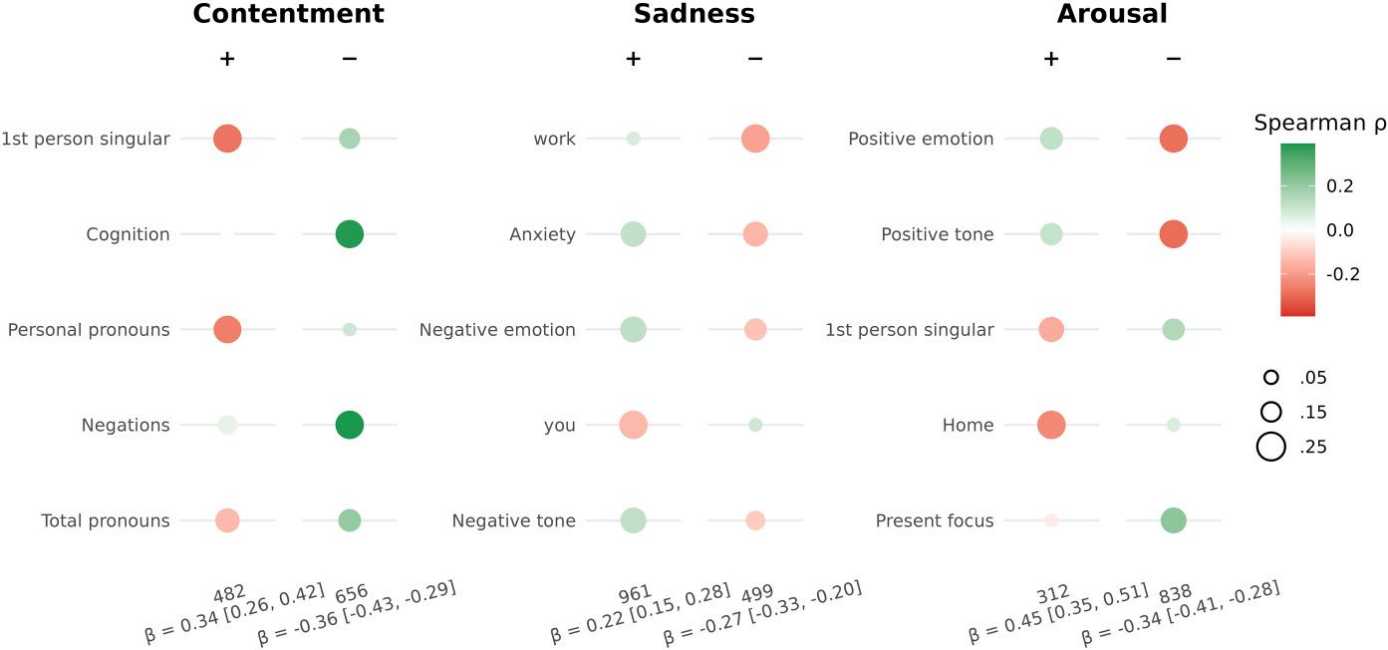
Spearman correlations of the most-predictive text-embedding dimensions for each emotional state with LIWC categories. For each emotional state (contentment, sadness, and arousal), the figure shows the embedding dimensions (indexed by their dimension number) with the largest average positive (+, left) and negative (−, right) regression weights (*β*), estimated using elastic-net models and averaged across 50 resampling iterations. Values in square brackets indicate the 95% interval of regression coefficients across resamplings. Dots indicate the correlations (Spearman's *ρ*) of the respective embedding dimensions with LIWC categories that were captured in the respective speech samples. Green reflects positive correlations, red reflects negative correlations, and dot sizes represent correlation magnitude. For each emotional state, we show those five LIWC categories that have the largest absolute difference in correlations between the positive and negative embedding dimensions and LIWC categories.

Given the superior predictive performance of variables derived from spoken content, we examined which linguistic variables were most predictive of emotional states across resamplings by analyzing coefficients from elastic-net models trained on LIWC word categories and text embeddings (Table 1 and Fig. 2). Across emotional states, the LIWC categories positive and negative emotional tone and positive and negative emotion words were consistently among the most important predictors. Positive emotion words (*β* = 0.05; 95% resampling interval [0.04, 0.05]) and positive tone (*β* = 0.02 [0.01, 0.02]) were linked to greater contentment and arousal (positive emotion: *β* = 0.03 [0.03, 0.04]; positive tone: *β* = 0.02 [0.01, 0.02]), whereas negative tone predicted lower contentment (*β* = −0.02 [−0.03, −0.02]) and arousal (*β* = −0.01 [−0.02, −0.01]) but higher sadness (*β* = 0.02 [0.01, 0.02]). Unsurprisingly, sadness words were especially predictive of higher sadness (*β* = 0.08 [0.07, 0.10]). More importantly, several nonemotion categories emerged as important predictors: the “want” category (e.g. “hope” and “wish”) was linked to lower contentment (*β* = −0.02 [−0.03, −0.01]) and slightly higher sadness (*β* = 0.00 [0.00, 0.01]), suggesting that desire-related language may capture unmet needs related to negative affect, while fatigue (e.g. “tired” and “bored”; *β* = −0.04 [−0.07, −0.03]) and health (e.g. “sick” and “medication”; *β* = −0.03 [−0.04, −0.01]) terms predicted lower arousal, potentially reflecting reduced energy states.

**Table 1:** Top five LIWC predictors of self-reported emotional states.

Contentment		Sadness		Arousal	
LIWC	*β* (95% interval)	LIWC	*β* (95% interval)	LIWC	*β* (95% interval)
Positive emotion	0.05 (0.04, 0.05)	Sadness	0.08 (0.07, 0.10)	Fatigue	−0.04 (−0.07, −0.03)
Want	−0.02 (−0.03, −0.01)	Positive emotion	−0.02 (−0.02, −0.02)	Positive emotion	0.03 (0.03, 0.04)
Negative tone	−0.02 (−0.03, −0.02)	Negative tone	0.02 (0.01, 0.02)	Health	−0.03 (−0.04, −0.01)
Positive tone	0.02 (0.01, 0.02)	Discrepancy	0.00 (0.00, 0.01)	Positive tone	0.02 (0.01, 0.02)
Discrepancy	−0.01 (−0.02, −0.01)	Want	0.00 (0.00, 0.01)	Negative tone	−0.01 (−0.02, −0.01)

The table reports the LIWC categories with the largest absolute mean coefficients (*β*) for each emotional state across 50 cross-validated elastic-net models, together with their 95% resampling interval. Positive coefficients indicate that higher word category use predicted higher self-reported state levels, whereas negative coefficients indicate lower predicted state levels.

We next identified the embedding dimensions that were most predictive for high and low levels of each emotional state. However, individual embedding dimensions are not readily interpretable. To link the abstract embedding space to psychologically meaningful linguistic constructs, we correlated each predictive embedding dimension with LIWC categories. Specifically, we report the five categories that best differentiate transcripts scoring high versus low on these embedding dimensions (Fig. 2). For instance, the embedding dimension most predictive of higher contentment (#482; *β* = 0.34; 95% resampling interval [0.26, 0.42]) was characterized by lower use of personal pronouns, especially first-person singular pronouns (e.g. “I” and “me”), along with reduced use of cognitive-process words and negations. The embedding dimension most predictive of lower contentment (#656; *β* = −0.36 [−0.43, −0.29]) showed the reverse pattern, consistent with a more self-focused way of talking about one's situation. This pattern aligns with prior work linking elevated first-person singular pronoun use to more negative emotional experience (21, 22). For sadness and arousal, the highly predictive dimensions systematically aligned with affective categories (e.g. positive and negative emotion words and emotional tone), which also emerged as important predictors in the LIWC models. This overlap in affective tone and emotionality between LIWC and predictive embedding dimensions may also explain why LIWC models performed similarly to those trained on text embeddings.

## Discussion

We find that brief, prompted, smartphone-captured speech samples, particularly their semantic content, contain information about people's self-reported emotional states in daily life, extending prior work on speech–emotion inference in the wild (11–14). Notably, these effects were obtained in naturalistic settings: we analyzed brief, heterogeneous in situ smartphone recordings that were automatically transcribed and paired with single-item self-reports of emotional states, a setting that poses a substantial challenge for inference. Performance levels (contentment: median Spearman *ρ* = 0.37; sadness: *ρ* = 0.24; arousal: *ρ* = 0.35) compare favorably with prior in-the-wild studies using concurrent emotion self-reports (11–14), which have typically reported modest associations under similar real-world conditions. For context, even more stable individual differences such as personality traits are only marginally better predicted from text and speech embeddings (up to *r* = 0.39; 16).

Across analyses, semantic content—“what” people said—carried the strongest emotional signal, indicating that emotional information in everyday speech is conveyed primarily through semantic properties. This prominence of semantic content in our results is consistent with theoretical accounts that assign language a central role in structuring and communicating emotional experience (5). This pattern should be interpreted in light of the study design: participants were prompted to describe their current situation, thoughts, and feelings, likely eliciting emotion-rich language that favors semantic representations. Within semantic approaches, modern text embeddings performed best, followed closely by LIWC-based variables. Masking emotion words disproportionately reduced LIWC performance relative to embeddings, consistent with the idea that dictionary-based methods rely more heavily on explicit affective vocabulary. Together, these findings suggest that LIWC remains a transparent and effective baseline for prompted, emotion-focused speech, whereas embedding-based approaches may be more effective at capturing emotional content when it is implicit, indirect, or context-dependent.

In contrast, traditional prosodic descriptors—capturing “how” people talked—conveyed little emotional signal in this heterogeneous real-world recording context. Prior work using naturalistic speech has likewise found that low-level acoustic cues often show limited predictive utility for momentary affective states in daily life (11, 14). One additional explanation is that device- and setting-related variability may have distorted vocal acoustics: differences in smartphone models, microphones, and recording distances could have degraded prosodic signals, impairing downstream inference (23). Therefore, in unconstrained, real-world settings, semantic information appears to be a more reliable carrier of subjective emotional states than low-level acoustic cues. Notably, speech embeddings derived directly from the audio signal performed comparably to text-based representations, likely because they encode not only acoustic properties but also aspects of semantic content (15, 24).

Together, these findings highlight the potential of brief speech samples as a scalable signal for studying subjective emotional states in everyday contexts. In research, smartphone-based speech inference could complement self-reports by enabling denser sampling of emotional dynamics with relatively low participant burden, consistent with recent work showing that active voice-based diary tools can be feasible in daily life (18, 19, 25). In applied settings, speech-derived indicators of emotional experience may support analyses of change over time and help contextualize individual differences, provided that models are carefully validated for the target population and use case. Future work could further improve inference by adopting idiographic or personalized modeling approaches that explicitly account for within-person variability in speech. This may be especially useful because speech reflects both stable speaker-specific characteristics and changing emotional states, which can limit models’ cross-person generalizability. Accordingly, personalized speech-based models may be a promising direction for improving prediction by better adapting to individual speakers (26, 27). Speech data are highly sensitive, so any deployment of mobile speech-based emotion inference must address ethical considerations, including privacy, informed consent, and the risk of misuse (28). To balance ecological validity with privacy protection, on-device processing approaches, where pretrained speech models extract embeddings or generate inferences locally, are particularly promising, minimizing the transmission of identifiable data (29). Such designs could support responsible research applications while preserving individual autonomy and data security.

### Limitations and future directions

Our findings should be interpreted in light of several constraints of the present study that also point to directions for future research. First, the generalizability of our results is bounded by the characteristics of the sample and by the assessed emotion outcomes. Participants were English-speaking young adults in the United States who voluntarily provided speech recordings. In addition, the present findings are specific to the particular emotion outcomes assessed here (contentment, sadness, and arousal) and to their operationalization using single-item self-report measures. Future studies should include more diverse linguistic, cultural, and socioeconomic populations as well as a broader and more differentiated set of emotional-state measures to assess the robustness of these findings across contexts.

Second, our findings apply to actively prompted speech, which differs in systematic ways from unprompted everyday conversation. The prompts were designed to elicit reflections on participants’ current experiences, likely increasing affective relevance relative to neutral or task-focused speech. At the same time, this design choice may have reduced ecological naturalness and encouraged recording in low-disruption contexts, potentially underrepresenting highly intense emotional situations. Passive data collection approaches using smartphones or other devices, such as the Electronically Activated Recorder, may capture more spontaneous speech but introduce distinct ethical and privacy challenges (13, 14, 30). Future work should therefore examine how collection modality (active vs. passive) and speech content jointly shape emotion inference, for example by comparing diary-style reflections with task-oriented dialogue.

Third, although our interpretability analyses linked predictive semantic representations to affective language categories, they offer only a partial view into how foundation models encode emotional information. Speech embeddings are particularly challenging to interpret because they entangle semantic and acoustic cues without a clear mapping to established linguistic categories. This challenge reflects a broader issue in representation learning: while foundation models capture rich information, their internal representations remain abstract and difficult to translate into theory. Future work should extend emerging interpretability approaches (31) to better characterize how emotional information is organized in embedding spaces and to assess the stability of such representations across contexts.

## Conclusion

In sum, brief smartphone-captured speech samples contain information about people's self-reported emotional states in everyday life. This finding highlights the potential of speech as a scalable signal for studying subjective emotional experience in naturalistic contexts, potentially complementing self-reports in settings where dense sampling is difficult. Distinguishing between emotional experience and emotional expression is essential for understanding what everyday speech can reveal about how people feel and for interpreting speech-based emotion inference in both research and applied contexts.

## Materials and methods

### Data collection

Data collection for the present work was part of the UT1000 Project at the University of Texas at Austin in the United States in fall 2018 (32). This research received approval from the University of Texas Institutional Review Board (study no. 2018-07-0035). During a 3-week self-tracking assignment, students in an introductory psychology Synchronous Massive Online Course used the Beiwe app (33), available on Android and iOS smartphones, to complete three short experience-sampling questionnaires per day (at 12, 3, and 6 PM), at the end of which they were asked to make a brief speech recording. All students included in this study provided informed consent for the use of their collected data. As part of the experience sampling, participants reported their momentary emotional states using single items: Contentment (“I am feeling content”) and sadness (“I am feeling sad”) were each rated on a 4-point Likert scale (0 = not at all, 1 = a little bit, 2 = quite a bit, and 3 = very much) intended to capture the presence and intensity of these emotional states. Arousal was operationalized as momentary energy level (“My energy level is”), to avoid potential ambiguity associated with the term “aroused” in everyday language, and was rated on a 5-point scale (0 = low energy, 1 = somewhat low energy, 2 = neutral, 3 = somewhat high energy, and 4 = high energy). Contentment and sadness were selected because they represent low-arousal affective states with opposite valence in the circumplex model of affect (34). Single-item measures of emotional states are widely used in experience-sampling research to minimize participant burden, yet they may be more susceptible to measurement error and therefore provide a less precise assessment than multi-item measures (35, 36). Given that the emotional-state items were assessed using different scale formats (4- and 5-point scales), comparisons across outcomes should be interpreted cautiously. Self-reported contentment scores were negatively correlated with sadness (*ρ* = −0.41) across all observations. Sadness was negatively correlated with arousal (*ρ* = −0.28), and contentment was positively correlated with arousal (*ρ* = 0.44). This positive contentment–arousal association suggests that “energy” may have captured everyday positive activation or vitality rather than arousal in the strict theoretical sense and that contentment may have been interpreted more broadly as general positive affect than as a strictly low-arousal state.

After completing each emotion report, participants were instructed to record a brief audio clip describing their current situation, thoughts, and feelings: “Please record a short audio clip in which you describe the situation you are in, and your current thoughts and feelings. Collect about 10 seconds of environmental sound after the description.” Only the spoken portions of each recording were analyzed; nonspeech segments at the beginning and end were later removed as part of the data processing. No additional denoising or noise-reduction preprocessing was applied prior to variable extraction, allowing us to analyze the recordings as captured by participants’ smartphones under naturalistic conditions.

We collected 21,027 speech samples from 977 participants (*M* = 21.52, SD = 17.30 speech samples per participant), each paired with emotion self-reports completed within 10 min of the recording. Figure S3 shows the distribution of voiced-speech duration and transcript length across recordings. We then removed 5,431 records containing fewer than 10 words or <5 s of voiced speech to exclude clips with extremely limited linguistic or acoustic information. These thresholds were chosen to ensure minimal usable signal for both transcript-based analyses and prosodic feature extraction while retaining as much naturalistic data as possible. We further examine model performance as a function of recording length in a sensitivity analysis (Fig. S2). Next, we dropped 3,311 samples in which a contemporary speaker diarization algorithm (NVIDIA Streaming Sortformer 4spk-v2), which detects whether more than one person is speaking in a recording, identified multiple speakers. This ensured that analyses were restricted to the participant's speech and excluded recordings containing other people talking or background sources, such as a TV. The final dataset comprised 12,285 speech samples (voiced-speech duration in seconds: *M* = 25.40, SD = 24.57, range = 5.04–238.72) averaging 57.36 words (SD = 54.55, range = 10–668) from 934 participants (63.65% female, mean age 18.59 years). Participants contributed an average of 13.15 recordings (SD = 11.29) with concurrent emotion self-reports: contentment: *M* = 1.65, SD = 0.86; sadness: *M* = 0.49, SD = 0.75; arousal: *M* = 1.95, SD = 0.95. We retained all available observations from each participant to make full use of the data, resulting in participants contributing different numbers of recordings. Distributions of all emotional-state ratings are shown in Fig. S1 and full demographic characteristics of the sample in Table S2.

### Variable extraction

We extracted four variable sets from speech samples (Table 2): text and speech embeddings derived from foundation models, as well as two types of traditional variables commonly used in speech analysis for emotion inference, namely dictionary-based word-use frequencies and prosodic voice descriptors (11, 13, 14).

**Table 2:** Overview of variable sets.

Variable set	Number of variables	Description
Text embeddings	1,024	Contextualized numeric representations of semantic content of transcripts from a pretrained large language model (RoBERTa-large)
Speech embeddings	1,024	Contextualized numeric representations derived from the speech signal using a pretrained speech foundation model (wav2vec2-large-robust-12-ft-emotion-msp-dim)
LIWC	99	Psychologically validated word-category frequencies from the latest version of the Linguistic Inquiry and Word Count (LIWC-22)
Prosodic descriptors	88	Low-level acoustic variables capturing pitch, intensity, rhythm, and related speech characteristics from the extended Geneva Minimalistic Acoustic Parameter Set (eGeMAPS)

We transcribed all audio recordings using a contemporary automatic speech recognition (ASR) model (NVIDIA Parakeet-TDT 0.6B-v2), which converts spoken audio into written text. Prior work suggests that ASR-generated transcripts can support downstream language analyses (37), although their quality depends on the specific ASR model and recording conditions. We then converted each transcript into a numerical representation (“embedding”) using the “text” R package (38). Specifically, we extracted token-level hidden states from the second-to-last (23rd) layer of the pretrained RoBERTa-large foundation model, consistent with prior work (39, 40). We then averaged these token-level representations across each transcript to obtain a single 1,024-dimensional embedding per transcript. From the transcripts, we also extracted 99 linguistic dimensions using the latest version of the LIWC-22 (41). LIWC is an established dictionary-based tool for language analysis that scans transcripts for words and stems (including multiword expressions), assigns them to psychologically interpretable categories (e.g. affect, social, and cognitive), and outputs per-category percentages relative to the total word count. We excluded summary variables, which are higher-order composites of the basic categories, as well as variables capturing punctuation.

We also passed each trimmed speech sample through a speech foundation model (wav2vec2-large-robust-12-ft-emotion-msp-dim), a large neural network trained to extract patterns from audio and fine-tuned for dimensional affect prediction (15, 42). From this model, we obtained 1,024-dimensional embeddings from the final transformer layer at each frame and then averaged these frame-level vectors to yield a single 1,024-dimensional embedding per audio recording. In addition, we extracted the extended Geneva Minimalistic Acoustic Parameter Set (eGeMAPS), consisting of 88 acoustic descriptors widely used in emotional speech analysis, including measures of pitch, loudness, spectral balance, formant characteristics, and voice quality, using the “openSMILE” Python library (43).

### Machine learning

We trained nonlinear tree-based random forest models, linear elastic net regularized regression models, and a baseline model on the extracted variables for the prediction of self-reported emotional states (contentment, sadness, and arousal). To fit the machine learning models, we employed the “mlr3” framework (44) in R and, more specifically, the “glmnet” package (45) and the “ranger” package (46). In the elastic-net models, we set the mixing parameter controlling the balance between LASSO and Ridge regularization to 0.5 for an equal mix. In the random forest models, we set the number of trees to 1,000. We predicted emotional states from all extracted variables combined and per variable set separately. The baseline model predicted the respective mean value for self-reported emotional states of the respective training set for all cases in a test set. We evaluated model performance by repeatedly training and testing the models on different subsets of the data (five repeats of 10-fold cross-validation). To ensure that performance reflected generalization to new individuals, all emotion–speech observations from a given participant were assigned entirely to either the training set or the test set in each resampling split (“blocking”). Consequently, performance reflects generalization to unseen participants rather than within-person tracking of emotional fluctuations. In the Results section, we report model performance across those 50 cross-validated models trained on all extracted variables and per variable group (text embeddings, speech embeddings, LIWC, and prosodic descriptors) in predicting emotional states (arousal, contentment, and sadness).

We evaluated model performance using the Spearman rank correlation (*ρ*) between self-reported and predicted emotion scores in participants whose data were not used for model training. In the supplements, we provide the out-of-sample coefficient of determination (*R*^2^), mean absolute error (MAE), and the root mean squared error (RMSE) as additional performance measures.

### Sensitivity analyses

We conducted three sensitivity analyses. First, to assess whether model performance relied on participants’ explicit verbalizations of their emotional state (e.g. “I am *happy*”) given that part of the instruction prompted them to talk about their current feelings, we conducted the analyses on the transcripts after masking common emotion words. Specifically, we used the 60 emotion adjectives of the PANAS-X (47), a widely used set of explicit emotion descriptors spanning fear, hostility, guilt, sadness, joviality, self-assurance, attentiveness, shyness, fatigue, serenity, and surprise. We complemented the list with eight manually added explicit emotion descriptors. Table S3 includes all terms used for masking. Each occurrence of these terms was replaced with the neutral token [EMO], approximately preserving the transcript's word count and surrounding syntax. Identical variable extraction, cross-validation splits, and model fitting were then applied to the masked transcript data. Second, to investigate whether model performance systematically varied with voiced-speech duration, we examined the relationship between the duration of voiced speech and prediction error. For elastic net models trained on all extracted variables combined, we extracted out-of-fold predictions from the resampling results for each emotional state (contentment, sadness, and arousal), computed absolute prediction error per observation (lower values indicate better model performance), and plotted those against voiced duration (Fig. S2). Third, because the text and speech embeddings each consisted of 1,024 variables, we applied PCA within the resampling procedure to reduce each embedding set to 100 components while retaining as much variance as possible to allow a more equal comparison with the lower-dimensional LIWC (99 variables) and eGeMAPS (88 variables) variable sets. Both elastic net and random forest models were then trained on these reduced embedding variables.

### Model interpretability

To gain insights into the linguistic characteristics of emotional speech, we examined the most-predictive LIWC variables and text-embedding dimensions in elastic-net models per emotional state. We used the regression coefficients (*β*), averaged across models as importance measures. For models trained on LIWC variables, we reported those five word categories with the largest absolute regression coefficients for each emotional state. For text embeddings, we identified dimensions with the largest positive and negative average regression coefficients for each emotional state. To enhance the interpretability of these predictive embedding dimensions, we computed Spearman correlations between each dimension and LIWC category scores across transcripts, thereby linking the abstract embedding space to psychologically meaningful linguistic constructs. We report the five LIWC categories that best differentiate these predictive dimensions (i.e. that show the largest difference in correlations).

### Use of artificial intelligence tools

We used OpenAI ChatGPT and Codex (models GPT-5.2, GPT-5.5, and GPT-5.6) to improve code scripts and make minor copy edits to human-generated text for readability and style. All outputs were reviewed and edited by the authors, who take full responsibility for the content.

## Supplementary Material

pgag263_Supplementary_Data

## Footnotes

1 For sadness, elastic net models trained on prosodic descriptors produced effectively constant predictions within folds (zero variance), yielding undefined Spearman rank correlations.

## Preprints

This manuscript was posted as a preprint: https://osf.io/preprints/psyarxiv/n48uz.

### Data Availability

All code used to reproduce the predictive analyses and detailed results are available in the project's OSF repository (https://osf.io/tha5x/). Raw speech samples may contain personally identifiable information and, therefore, cannot be shared. Because derived features (i.e. embeddings, LIWC scores, and prosodic descriptors) may still carry privacy risks, they will be shared with qualified researchers upon reasonable request.
