# Supplementary material for: Brief speech samples reveal emotional states in daily life
Source: PNAS Nexus. 2026 Aug 6;5(8):pgag263. doi: 10.1093/pnasnexus/pgag263 (PMC13458938; doi:10.1093/pnasnexus/pgag263)
Supplement: Supplementary file 1 [file pgag263_supplementary_data.pdf]

PNAS nexus

Supplementary Information for
Brief Speech Samples Reveal Emotional States in Daily Life

Timo K. Koch, Gabriella M. Harari, Samuel D. Gosling, Zachariah Marrero, Ramona Schoedel, Markus Bühner, & Clemens Stachl

Timo K. Koch
Email: timo.koch@unisg.ch

This PDF file includes:

Figures S1 to S3
Tables S1 to S3
SI References

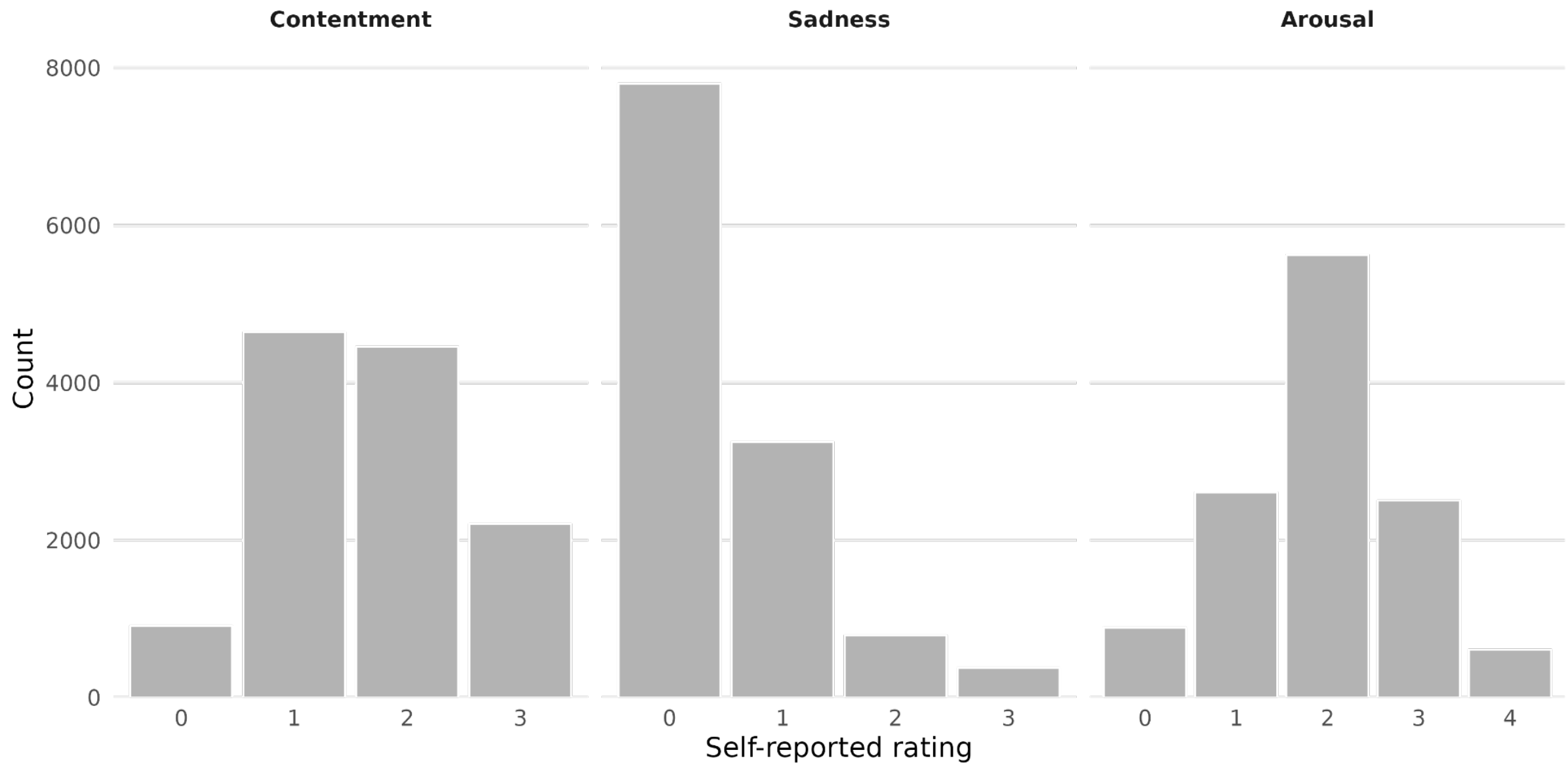

Fig. S1. Bar plots show the frequency of self-report ratings for contentment, sadness, and arousal across all retained speech recordings. Contentment and sadness were rated on a 0–3 scale, whereas arousal was rated on a 0–4 scale.

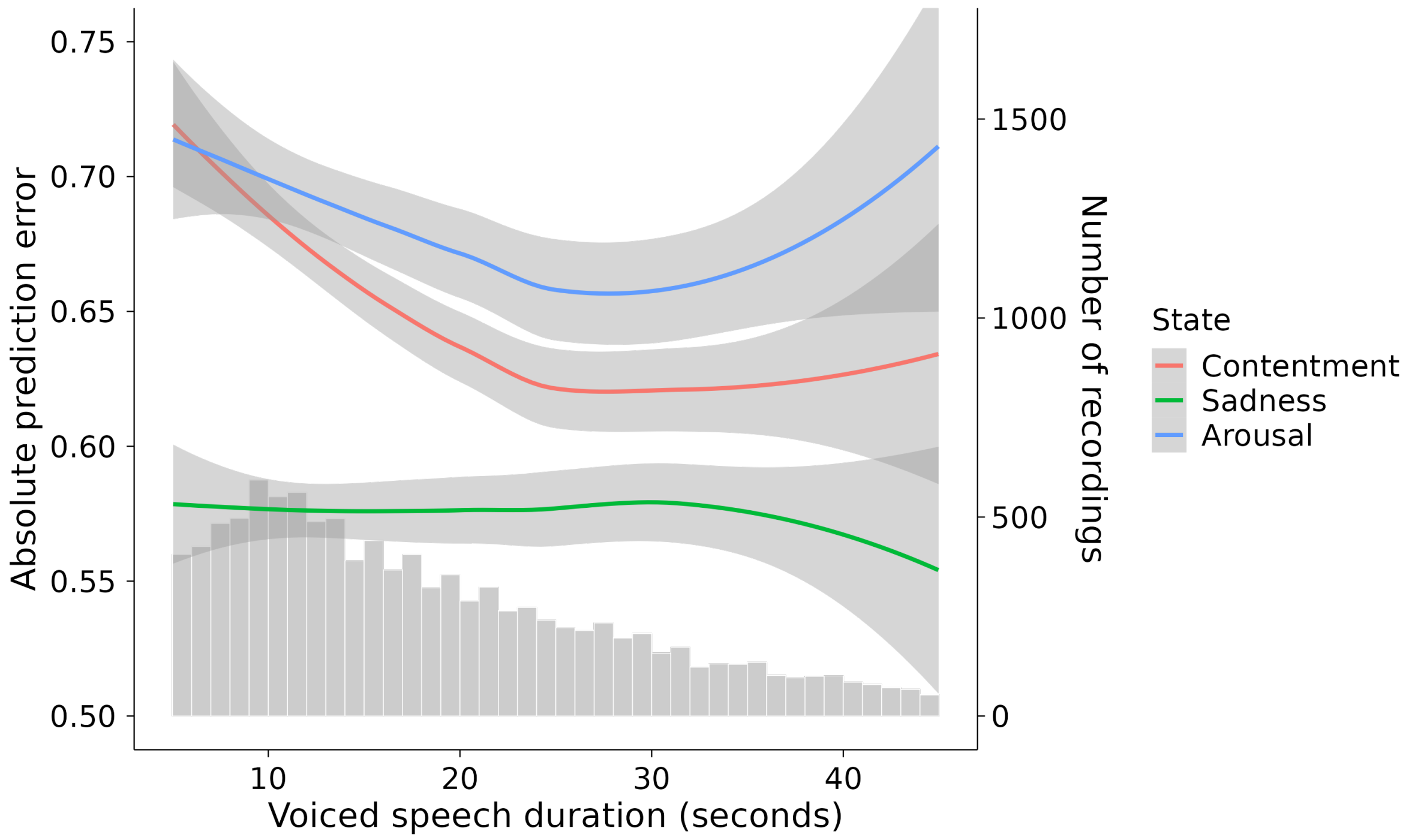

Fig. S2. Lines show LOESS-smoothed relationships between voiced speech duration (x-axis, in seconds) and absolute prediction error (y-axis) for five times repeated tenfold cross-validated elastic net ensemble models combining all extracted variables to estimate emotional states (contentment, sadness, and arousal). Shaded bands indicate standard errors around the LOESS smooths. We averaged absolute prediction error across repeated out-of-fold predictions for each recording and emotional state, then examined its association with voiced speech duration. Grey background bars show the distribution of voiced speech duration across recordings. The plot and LOESS fits are restricted to recordings with 5–45 seconds of voiced speech.

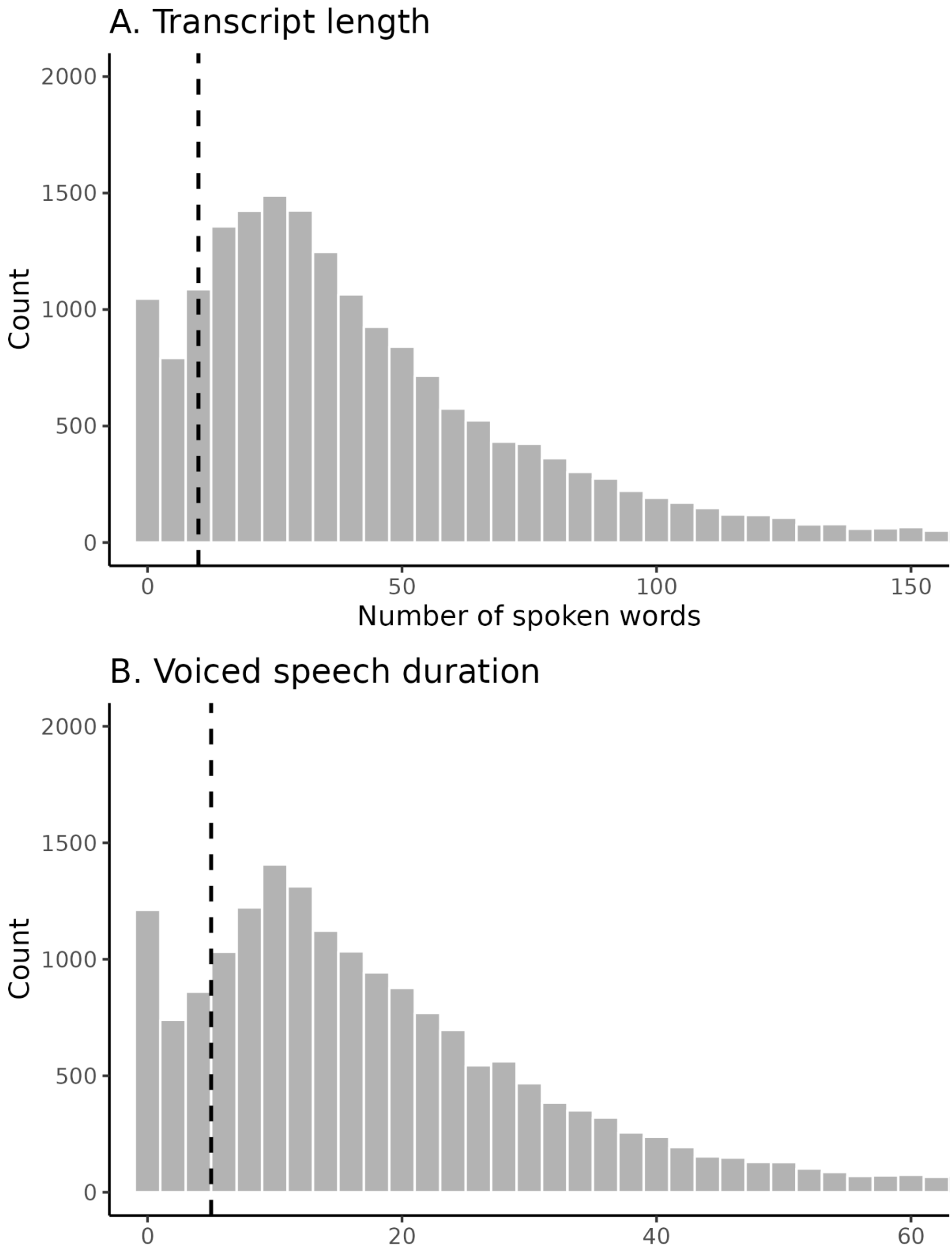

Fig. S3. Histograms showing the distribution of speech sample length across all recordings with available EMA data. Panel A displays transcript length in spoken words, and Panel B displays voiced speech duration in seconds. Dashed vertical lines indicate the minimum inclusion thresholds of 10 spoken words and 5 seconds of voiced speech, respectively. For readability, the displayed ranges are limited to 0–160 spoken words and 0–60 seconds of voiced speech; recordings exceeding these limits are not shown.

Table S1. Prediction Performance Results.

Outcome Variable	Predictor Variables	Algo.	ρ [95% interval]	R^2 [95% interval]	*MAE* [95% interval]	*RMSE* [95% interval]
Contentment	All	FL	NA [NA, NA]	-0.004 [-0.053, -0.000]	0.739 [0.689, 0.797]	0.856 [0.798, 0.925]
Contentment	All	EN	0.366 [0.318, 0.421]	0.128 [0.051, 0.169]	0.658 [0.612, 0.715]	0.796 [0.737, 0.870]
Contentment	All	RF	0.349 [0.292, 0.412]	0.119 [0.055, 0.158]	0.669 [0.622, 0.726]	0.800 [0.741, 0.868]
Contentment	Text embeddings	FL	NA [NA, NA]	-0.004 [-0.053, -0.000]	0.739 [0.689, 0.797]	0.856 [0.798, 0.925]
Contentment	Text embeddings	EN	0.355 [0.281, 0.399]	0.118 [0.046, 0.150]	0.669 [0.616, 0.731]	0.804 [0.742, 0.876]
Contentment	Text embeddings (PCA)	EN	0.359 [0.285, 0.413]	0.118 [0.049, 0.144]	0.674 [0.620, 0.731]	0.803 [0.743, 0.873]
Contentment	Text embeddings	RF	0.342 [0.273, 0.392]	0.101 [0.053, 0.120]	0.687 [0.630, 0.741]	0.814 [0.745, 0.873]
Contentment	Text embeddings (PCA)	RF	0.348 [0.279, 0.405]	0.096 [0.043, 0.120]	0.692 [0.634, 0.746]	0.814 [0.748, 0.875]
Contentment	Text embeddings (masked)	FL	NA [NA, NA]	-0.004 [-0.053, -0.000]	0.739 [0.689, 0.797]	0.856 [0.798, 0.925]
Contentment	Text embeddings (masked)	EN	0.331 [0.246, 0.378]	0.102 [0.028, 0.131]	0.679 [0.626, 0.741]	0.809 [0.751, 0.886]
Contentment	Text embeddings (masked)	RF	0.318 [0.241, 0.373]	0.086 [0.035, 0.107]	0.694 [0.640, 0.750]	0.819 [0.755, 0.883]
Contentment	Speech embeddings	FL	NA [NA, NA]	-0.004 [-0.053, -0.000]	0.739 [0.689, 0.797]	0.856 [0.798, 0.925]
Contentment	Speech embeddings	EN	0.319 [0.252, 0.376]	0.100 [0.026, 0.141]	0.671 [0.628, 0.737]	0.810 [0.750, 0.885]
Contentment	Speech embeddings (PCA)	EN	0.313 [0.250, 0.373]	0.097 [0.025, 0.129]	0.679 [0.635, 0.739]	0.813 [0.755, 0.881]
Contentment	Speech embeddings	RF	0.311 [0.251, 0.371]	0.099 [0.028, 0.134]	0.677 [0.631, 0.738]	0.809 [0.753, 0.881]
Contentment	Speech embeddings (PCA)	RF	0.308 [0.244, 0.379]	0.082 [0.022, 0.113]	0.695 [0.646, 0.754]	0.821 [0.758, 0.890]
Contentment	LIWC	FL	NA [NA, NA]	-0.004 [-0.053, -0.000]	0.739 [0.689, 0.797]	0.856 [0.798, 0.925]
Contentment	LIWC	EN	0.331 [0.246, 0.376]	0.092 [0.035, 0.115]	0.690 [0.638, 0.742]	0.817 [0.754, 0.876]
Contentment	LIWC	RF	0.337 [0.251, 0.388]	0.110 [0.050, 0.141]	0.671 [0.628, 0.726]	0.807 [0.749, 0.869]
Contentment	LIWC (masked)	FL	NA [NA, NA]	-0.004 [-0.053, -0.000]	0.739 [0.689, 0.797]	0.856 [0.798, 0.925]
Contentment	LIWC (masked)	EN	0.282 [0.202, 0.338]	0.064 [0.010, 0.089]	0.705 [0.648, 0.755]	0.828 [0.764, 0.891]
Contentment	LIWC (masked)	RF	0.295 [0.206, 0.345]	0.081 [0.028, 0.110]	0.690 [0.636, 0.742]	0.821 [0.756, 0.880]
Contentment	eGeMAPS	FL	NA [NA, NA]	-0.004 [-0.053, -0.000]	0.739 [0.689, 0.797]	0.856 [0.798, 0.925]
Contentment	eGeMAPS	EN	0.150 [0.040, 0.197]	0.014 [-0.038, 0.026]	0.730 [0.678, 0.787]	0.850 [0.791, 0.917]
Contentment	eGeMAPS	RF	0.138 [0.071, 0.214]	0.013 [-0.039, 0.042]	0.725 [0.660, 0.781]	0.849 [0.785, 0.917]
Sadness	All	FL	NA [NA, NA]	-0.005 [-0.046, -0.000]	0.617 [0.574, 0.709]	0.743 [0.652, 0.898]

Sadness	All	EN	0.239 [0.174, 0.300]	0.075 [0.020, 0.140]	0.576 [0.529, 0.667]	0.710 [0.627, 0.871]
Sadness	All	RF	0.181 [0.093, 0.257]	0.050 [-0.019, 0.097]	0.596 [0.556, 0.688]	0.724 [0.644, 0.871]
Sadness	Text embeddings	FL	NA [NA, NA]	-0.005 [-0.046, -0.000]	0.617 [0.574, 0.709]	0.743 [0.652, 0.898]
Sadness	Text embeddings	EN	0.233 [0.158, 0.280]	0.066 [0.024, 0.108]	0.585 [0.538, 0.665]	0.715 [0.631, 0.862]
Sadness	Text embeddings (PCA)	EN	0.227 [0.151, 0.298]	0.057 [0.019, 0.096]	0.589 [0.543, 0.672]	0.717 [0.633, 0.867]
Sadness	Text embeddings	RF	0.146 [0.037, 0.218]	0.020 [-0.059, 0.062]	0.612 [0.575, 0.692]	0.735 [0.659, 0.872]
Sadness	Text embeddings (PCA)	RF	0.160 [0.082, 0.217]	0.030 [-0.032, 0.071]	0.608 [0.572, 0.692]	0.726 [0.654, 0.871]
Sadness	Text embeddings (masked)	FL	NA [NA, NA]	-0.005 [-0.046, -0.000]	0.617 [0.574, 0.709]	0.743 [0.652, 0.898]
Sadness	Text embeddings (masked)	EN	0.196 [0.125, 0.250]	0.042 [-0.003, 0.078]	0.594 [0.551, 0.679]	0.723 [0.641, 0.871]
Sadness	Text embeddings (masked)	RF	0.125 [0.021, 0.207]	0.004 [-0.075, 0.051]	0.614 [0.582, 0.697]	0.740 [0.665, 0.875]
Sadness	Speech embeddings	FL	NA [NA, NA]	-0.005 [-0.046, -0.000]	0.617 [0.574, 0.709]	0.743 [0.652, 0.898]
Sadness	Speech embeddings	EN	0.196 [0.123, 0.260]	0.046 [0.003, 0.099]	0.587 [0.536, 0.675]	0.724 [0.634, 0.880]
Sadness	Speech embeddings (PCA)	EN	0.186 [0.111, 0.259]	0.042 [-0.010, 0.086]	0.594 [0.545, 0.683]	0.724 [0.634, 0.881]
Sadness	Speech embeddings	RF	0.168 [0.077, 0.249]	0.038 [-0.035, 0.087]	0.599 [0.562, 0.694]	0.725 [0.649, 0.876]
Sadness	Speech embeddings (PCA)	RF	0.148 [0.031, 0.250]	0.024 [-0.033, 0.073]	0.606 [0.568, 0.701]	0.727 [0.648, 0.882]
Sadness	LIWC	FL	NA [NA, NA]	-0.005 [-0.046, -0.000]	0.617 [0.574, 0.709]	0.743 [0.652, 0.898]
Sadness	LIWC	EN	0.220 [0.132, 0.267]	0.041 [-0.020, 0.072]	0.597 [0.555, 0.689]	0.724 [0.641, 0.880]
Sadness	LIWC	RF	0.198 [0.128, 0.267]	0.057 [-0.016, 0.101]	0.588 [0.553, 0.679]	0.722 [0.641, 0.866]
Sadness	LIWC (masked)	FL	NA [NA, NA]	-0.005 [-0.046, -0.000]	0.617 [0.574, 0.709]	0.743 [0.652, 0.898]
Sadness	LIWC (masked)	EN	0.154 [0.073, 0.227]	0.012 [-0.031, 0.036]	0.609 [0.566, 0.696]	0.733 [0.647, 0.886]
Sadness	LIWC (masked)	RF	0.134 [0.054, 0.184]	0.013 [-0.049, 0.048]	0.609 [0.571, 0.693]	0.729 [0.659, 0.878]
Sadness	eGeMAPS	FL	NA [NA, NA]	-0.005 [-0.046, -0.000]	0.617 [0.574, 0.709]	0.743 [0.652, 0.898]
Sadness	eGeMAPS	EN	NA [NA, NA]	-0.005 [-0.046, -0.000]	0.617 [0.574, 0.709]	0.743 [0.652, 0.898]
Sadness	eGeMAPS	RF	0.019 [-0.084, 0.107]	-0.035 [-0.098, -0.001]	0.625 [0.584, 0.722]	0.754 [0.669, 0.912]
Arousal	All	FL	NA [NA, NA]	-0.002 [-0.023, -0.000]	0.678 [0.601, 0.785]	0.943 [0.869, 1.035]
Arousal	All	EN	0.352 [0.277, 0.395]	0.120 [0.069, 0.161]	0.681 [0.619, 0.762]	0.885 [0.812, 0.962]
Arousal	All	RF	0.349 [0.281, 0.384]	0.121 [0.067, 0.155]	0.677 [0.608, 0.759]	0.885 [0.811, 0.965]

Arousal	Text embeddings	FL	NA [NA, NA]	-0.002 [-0.023, -0.000]	0.678 [0.601, 0.785]	0.943 [0.869, 1.035]
Arousal	Text embeddings	EN	0.322 [0.245, 0.382]	0.097 [0.052, 0.128]	0.682 [0.616, 0.772]	0.899 [0.825, 0.974]
Arousal	Text embeddings (PCA)	EN	0.320 [0.254, 0.371]	0.091 [0.057, 0.121]	0.685 [0.615, 0.771]	0.900 [0.826, 0.978]
Arousal	Text embeddings	RF	0.314 [0.249, 0.380]	0.084 [0.051, 0.110]	0.677 [0.602, 0.771]	0.901 [0.829, 0.988]
Arousal	Text embeddings (PCA)	RF	0.318 [0.253, 0.366]	0.081 [0.041, 0.104]	0.675 [0.599, 0.770]	0.905 [0.829, 0.989]
Arousal	Text embeddings (masked)	FL	NA [NA, NA]	-0.002 [-0.023, -0.000]	0.678 [0.601, 0.785]	0.943 [0.869, 1.035]
Arousal	Text embeddings (masked)	EN	0.293 [0.215, 0.350]	0.079 [0.034, 0.105]	0.687 [0.620, 0.777]	0.911 [0.834, 0.989]
Arousal	Text embeddings (masked)	RF	0.281 [0.220, 0.351]	0.070 [0.039, 0.091]	0.679 [0.606, 0.773]	0.909 [0.836, 0.993]
Arousal	Speech embeddings	FL	NA [NA, NA]	-0.002 [-0.023, -0.000]	0.678 [0.601, 0.785]	0.943 [0.869, 1.035]
Arousal	Speech embeddings	EN	0.323 [0.255, 0.374]	0.105 [0.045, 0.145]	0.688 [0.623, 0.771]	0.896 [0.824, 0.974]
Arousal	Speech embeddings (PCA)	EN	0.317 [0.244, 0.361]	0.098 [0.048, 0.132]	0.689 [0.618, 0.773]	0.897 [0.821, 0.979]
Arousal	Speech embeddings	RF	0.329 [0.262, 0.362]	0.108 [0.058, 0.142]	0.683 [0.615, 0.766]	0.891 [0.819, 0.972]
Arousal	Speech embeddings (PCA)	RF	0.313 [0.252, 0.358]	0.087 [0.052, 0.116]	0.679 [0.606, 0.770]	0.897 [0.825, 0.985]
Arousal	LIWC	FL	NA [NA, NA]	-0.002 [-0.023, -0.000]	0.678 [0.601, 0.785]	0.943 [0.869, 1.035]
Arousal	LIWC	EN	0.291 [0.231, 0.346]	0.064 [0.015, 0.095]	0.681 [0.603, 0.768]	0.916 [0.835, 0.989]
Arousal	LIWC	RF	0.321 [0.262, 0.380]	0.104 [0.061, 0.143]	0.682 [0.612, 0.756]	0.893 [0.821, 0.962]
Arousal	LIWC (masked)	FL	NA [NA, NA]	-0.002 [-0.023, -0.000]	0.678 [0.601, 0.785]	0.943 [0.869, 1.035]
Arousal	LIWC (masked)	EN	0.233 [0.159, 0.274]	0.039 [0.012, 0.061]	0.690 [0.613, 0.782]	0.927 [0.848, 1.009]
Arousal	LIWC (masked)	RF	0.248 [0.180, 0.302]	0.057 [0.027, 0.084]	0.693 [0.620, 0.782]	0.919 [0.843, 0.996]
Arousal	eGeMAPS	FL	NA [NA, NA]	-0.002 [-0.023, -0.000]	0.678 [0.601, 0.785]	0.943 [0.869, 1.035]
Arousal	eGeMAPS	EN	0.105 [0.032, 0.197]	0.005 [-0.015, 0.020]	0.679 [0.602, 0.786]	0.939 [0.863, 1.029]
Arousal	eGeMAPS	RF	0.113 [0.034, 0.187]	0.008 [-0.025, 0.037]	0.703 [0.635, 0.801]	0.933 [0.867, 1.026]

Out-of-sample prediction performance across algorithms and prediction tasks with multiple performance measures in a five times repeated ten-fold cross-validation scheme. FL = featureless (baseline) learner; EN = elastic net; RF = random forest. If predictions are constant within a fold, Spearman’s ρ is undefined and is therefore reported as NA.

Table S2. Demographic composition of the final sample.

N (participants)	934
N (observations)	12,285
Age, mean (*SD*)	18.59 (1.22)
Gender: Female (%)	63.65
Gender: Male (%)	36.02
Gender: Other (%)	0.33
Ethnicity: White (%)	33.52
Ethnicity: Asian/Asian American (%)	27.54
Ethnicity: Hispanic/Latino (%)	20.35
Ethnicity: Multiple (%)	13.05
Ethnicity: Other (%)	5.53

Demographic values are reported at the participant level. For ethnicity, “Multiple” indicates participants who selected more than one ethnic category.

Table S3. Emotion words used for lexical masking in speech transcripts.

Source	Words
PANAS-X (60)	active; afraid; alert; alone; amazed; angry; angry at self; ashamed; astonished; at ease; attentive; bashful; blameworthy; blue; bold; calm; cheerful; concentrating; confident; daring; delighted; determined; disgusted; disgusted with self; dissatisfied with self; distressed; downhearted; drowsy; energetic; enthusiastic; excited; fearless; frightened; guilty; happy; hostile; inspired; interested; irritable; jittery; joyful; lively; loathing; lonely; nervous; proud; relaxed; sad; scared; scornful; shaky; sheepish; shy; sleepy; sluggish; strong; surprised; timid; tired; upset
Manually added (8)	anxious; burned out; chill; depressed; exhausted; overwhelmed; stressed; worried

List of all emotion state terms used for masking, combining the 60 PANAS-X (Positive and Negative Affect Schedule–Expanded Form; *1*) adjectives with eight manually added common everyday emotion state words. Words are shown in alphabetical order within each row.

SI References

1. D. Watson, L. Clark, The PANAS-X: Manual for the positive and negative affect schedule-expanded form. *Iowa Res. Online* (1994).
